# It’s not just about how long you play. Indirect gaming involvement and genre preferences in predicting gaming disorder risk: evidence from preregistered studies

**DOI:** 10.3389/fpsyt.2023.1230774

**Published:** 2023-09-18

**Authors:** Paweł Strojny, Patrycja Kiszka, Jolanta Starosta, Paulina Daria Szyszka, Sylwia Starzec, Anna Winiarska, Agnieszka Strojny, Aleksandra Zajas

**Affiliations:** ^1^Institute of Applied Psychology, Faculty of Management and Social Communication, Jagiellonian University, Kraków, Poland; ^2^Institute of Psychology, Faculty of Philosophy, Jagiellonian University, Kraków, Poland; ^3^Doctoral School in the Social Sciences, Jagiellonian University, Kraków, Poland

**Keywords:** video games, gaming disorder, gaming involvement, video game genres, gaming motivation

## Abstract

**Introduction:**

The strength of the association between gaming involvement and gaming disorder is weak to moderate. Gamers cannot be directly involved in gaming all the time, but how much they are involved in activities indirectly related to gaming during gaming-free time may play an important role. Also, specific game genres may matter. The present investigation focuses on the role of indirect gaming involvement and genres in gaming disorder risk prediction.

**Methods:**

Two pre-registered studies were conducted. Study 1 (*N* = 205) was conducted online, whereas Study 2 (*N* = 250) was conducted in a lab. In both cases, participants reported their direct and indirect involvement in gaming (DGI and IGI, respectively) and completed a screening tool that estimates the risk of gaming disorder (Gaming Disorder Test).

**Results:**

Both IGI and DGI were weakly to moderately correlated with gaming disorder (GD) and moderately with each other. The correlations between DGI and GD were similar to those obtained in related studies; the correlation between IGI and GD has not been previously reported. Hierarchical regression that took IGI together with DGI into account showed an increase in the percentage of explained variance, but only in Study 1. Contrary to expectations, IGI did not interact with DGI. As is consistent with previous research, some game genres were found to be more closely related to GD than others: in both studies, this was an RPG; in Study 1, this was also an MMORPG; in Study 2, driving and shooting games also predicted GD risk.

**Discussion:**

Overall, the results clearly indicate that not only gaming time plays a role in GD risk assessment: IGI can also predict it and in some cases may allow for more accurate predictions. Gaming genres once again proved to play a role, but these and similar results should be treated with caution due to the partial lack of repeatability.

## Introduction

The casual association between gaming intensity and gaming disorder can lead to oversimplification and potential stigma against gamers and the gaming industry (1). It is important to note that the majority of video gamers do not experience gaming disorder or experience adverse consequences from gaming (2–6). However, gaming involvement is considered to be one of the most important variables with regards to gaming disorder. The strength of the correlation ranges from weak to moderate, with correlation coefficients between 0.17 and 0.40 (4, 7–10). Consequently, the assertion made in Orsolya Király and colleagues’ study title that “Intense video gaming is not essentially problematic” still appears to hold true (4). It follows that the focus should be on finding moderators of this association, so the current work focuses on this issue and reports on two pre-registered studies.

Gaming Involvement (GI) alone is not considered a reliable predictor of GD, as the studies cited above show that it is only moderately, at most, associated with gaming disorder (GD) (11). Additionally, Pontes et al. (12) challenged the widely accepted notion that disordered gaming occurs after approximately 30 h of weekly gaming. Their findings demonstrated that individuals who fulfill the disordered gaming criteria surpassed this threshold, indicating that relying solely on time spent gaming as an indicator of GD may be insufficient. These insights emphasize the importance of considering functional impairment and other indicators when investigating GD risk. Drawing upon the significant contributions of Pontes et al. (12), our current study aims to explore contextual factors that may moderate the basic “dosage effect” (13). This is in line with Demetrovics and Király’s (3) conclusions that researchers should not ignore the motives, consequences, and contextual characteristics of problematic gaming behavior when looking for a simple relationship between time spent on gaming and gaming disorder. We identified two factors that could moderate the effect size of the dosage effect. The first contextual factor is the range and intensity of activities indirectly related to gaming, which may or may not accompany gaming itself; the second factor is game genres. While both factors have been studied in the past, the work done so far has not led to clear conclusions, therefore we decided to investigate both gaming involvement and game genres in the two pre-registered studies presented below.

Observing the patterns of gaming behavior over the years, one can notice a gradual change in the proportion of gaming itself to other gaming-related activities. These activities can be understood as thinking about gaming, communicating about gaming, watching/listening to materials about games, or shopping for goods related to gaming. Two or three decades ago, gaming itself was the main free-time activity of gamers, perhaps because of the relatively difficult access to other game-related materials or communities ready to exchange experiences or strategies. This may not still be true today, when gamers devote their time to not only gaming but also other activities indirectly related to it. Therefore, limiting contemporary research to only asking participants about “the number of hours spent playing video games per week” (7, p. 6) or “weekly time spent gaming” (9, p. 2) may lead to incomplete results. Studies on some of the aforementioned activities have recently been published by Cabreza-Ramirez et al. (14, 15), Ortiz de Gortari and Panagiotidi (16), and Yildiz Durak et al. (17); however, the results seem to be mixed.

While Ortiz de Gortari and Panagiotidi’s (16) study revealed that intrusive gaming thoughts may be correlated with GD, relations between GD and time spent watching game-related content on streaming platforms (e.g., Twitch) appear to be more complicated. Cabreza-Ramirez et al. (14) showed that perceived positive uses of streaming platforms (e.g., making friends) and motivations for watching content on streaming platforms (e.g., sense of belonging) may be connected to key components of pathological game use. At the same time, no direct connection between time spent watching streams and pathological game use was detected. Another study by Cabreza-Ramirez et al. (15) yielded similar results: positive perceptions of gaming and certain motivations for using streaming services were associated with both pathological game use and time spent watching streams, even though no connection was found directly between time spent watching streams and problematic game use. Interestingly, Yildiz Durak et al. (17) showed that while time spent watching Twitch was associated with GD, time spent viewing streaming in general was not.

Thus, for the sake of our studies, we assumed that the operationalization of GI needed to be broadened. We decided to distinguish between activities directly related to gaming (Direct Gaming Involvement, DGI) and activities indirectly related to gaming (Indirect Gaming Involvement, IGI), exploring not only thinking about video games or watching game-related videos, but also other aspects of IGI. We expected that DGI would be positively and moderately correlated with GD risk (H1). We also expected to find a positive correlation between IGI and GD risk, but we did not hypothesize on the strength of this relationship (H2). Since IGI content can capture aspects of gaming behavior that cannot be captured by DGI, it was also expected that including IGI in the hierarchical multiple regression model along with DGI would explain more variance of GD risk than DGI alone (H3).

In addition, according to the I-PACE model, third variables can increase the level of gratification from gaming (18) and thus moderate the risk of GD. Previous studies have provided reasons to support these expectations. For example, Yu et al. (13) showed that social factors (e.g., loneliness, social anxiety) and maladaptive cognition play the role of moderators, while Koncz et al. (11) showed a significant role of depressive symptoms, ADHD, and specific motivation (escapism). These results suggest that gaming itself (DGI) is not harmful and that it can only provide an accurate idea of GD risk when analyzed in a broader context. As previous works focused on testing the moderating role of mental states, we asked whether it was possible for observable behaviors to play a similar role.

According to the updated I-PACE model (19), environmental factors (e.g., availability, affordability, reinforcement distribution) can play a role in the process of GD development. Therefore, we hypothesized that behaviors directly related to gaming would extend the spectrum of environmental factors affecting the user. When gaming is difficult or impossible for objective reasons (e.g., at work), other gaming-related activities may provide users with gratification. In consequence, they may spend a great percentage of their time on such activities, ultimately leading them to GD. As shown by Cabeza-Ramirez and colleagues (14), DGI is associated with IGI (in this case: time spent watching game-related streams), and, as these authors suggest, depending on the prioritization of each activity, the effects on GD may vary. Thus, both DGI and IGI should be explored.

One would expect that since these other activities not only prolong the amount of time spent in contact with gaming but also provide gratification of a broader nature, the extension of gaming time should manifest itself in mutually reinforcing the association between IGI and DGI. This is why we expected a difference in GD risk between two people with similar gaming intensity but different amounts of time spent on other activities related to gaming. We expected that high DGI combined with high IGI that manifests as activities such as thinking about or discussing gaming, consuming game-related content, and making gaming purchases may be a stronger indicator of GD than an equally high DGI without the accompanying IGI. As a consequence, we hypothesized that behavior indirectly related to gaming (IGI) can act as a moderator of the dosage effect (H4).

Furthermore, IGI implies that preoccupation is one of the clinical symptoms of internet gaming disorder mentioned in DSM-5 (20) and Griffith’s definition of behavioral addiction (21). This symptom may be understood as cognitive salience or obsessive thoughts regarding gaming activities (22). However, DSM-5 emphasizes that preoccupation reflects the cognitive priority of games in the mind of the player during not only gaming time but also non-gaming time (23), which may take the indirect form of thinking about game strategies, watching game-related videos, buying game-related items, etc. In other words, we expected an interaction between DGI and IGI for GD (H4). To our knowledge, no one has previously investigated IGI in terms of the amount of time spent on all the aforementioned indirectly gaming-related activities, so our data will contribute to the exploration of this area.

The moderating role of game genres has been explored before. We reasoned that the characteristics of individual game genres are so varied that they should result in certain game genres being disproportionately associated with GD. In previous studies, certain game genres were indeed more strongly related to GD, but the results were not consistent. For instance, studies have indicated that certain game genres, such as first-person shooters (FPS), action-adventures, or massively multiplayer online role-playing games (MMORPG), are more strongly related to GD (24). However, others have shown the association between GD and RPGs (25), or between GD and both real-time strategies (RTS) and RPGs (26). Moreover, some studies have identified links between problematic gaming and genres like RPGs, action-adventures, shooters, and sports games (27), RPGs, simulations, and Multiplayer Online Battle Arena (MOBA) games (28), or MMORPGs and shooters (29). Research on genres did not provide clear results, but we expected to replicate previous results: time spent on RPGs, RTSs, MMORPGs, shooters, and action games should predict GD risk (H5). Additionally, we expected that card games would also predict GD risk (H5).

We hypothesized that certain game genres might be particularly relevant to the development of GD due to their specific gameplay mechanics and psychological appeal. For instance, RPGs often feature immersive and continuous progress systems, which can foster a sense of achievement and reward, potentially leading to prolonged gaming sessions. Shooters, on the other hand, emphasize competition and social interaction, making them more engaging for extended periods. RTS games may involve complex decision-making and planning, which could lead to excessive time investment. MMORPGs often provide extensive social communities and a persistent virtual world, thus fostering long-term engagement. In addition to the aforementioned genres, we also propose that action games and card games could be associated with GD risk. Action games are known for their fast-paced and adrenaline-inducing gameplay, which might contribute to extended play. Card games, despite being a relatively new genre in the context of GD research, often employ mechanics that encourage players to log in regularly, potentially leading to compulsive gaming behavior. Additionally, one aspect that caught our attention and motivated our hypothesis is the prevalence of loot boxes in certain game genres, including RPGs, RTS, MMORPGs, shooters, action games and card games. Loot boxes, which are virtual items containing randomized rewards, have been a subject of growing concern due to their potential association with addictive gaming behaviors and their resemblance to gambling mechanics (e.g., 30, 31).

To verify each of the hypotheses, we conducted two studies that were complementary in terms of methodological decisions.

## Methods – Study 1

### Participants and procedure

Participants were recruited via a Polish online research platform called SWPanel. The survey was available to all registered users. Before the survey began, the participants gave their informed consent. Next, a preselection was made with one screening question: “Have you played video games (on your phone, computer, tablet, or any electronic device) in the last two weeks?”. Subjects who provided a positive answer were allowed to participate in the study. Then, they were asked to complete several self-report scales. The whole procedure took about 8 min. All participants were compensated. The reward for filling out the survey was “points,” which are exchangeable for money. The exact number of points was not disclosed by the company.

The planned sample size was *N* = 162, calculated according to Tabachnick et al. (32): given that a multiple regression model would involve 14 predictors, *N* = 50 + 8* 14 = 162. We initially collected a total sample of 620 observations between October 28 and November 14, 2022. We excluded participants for the following reasons: had not played any games in the previous 2 weeks (*n* = 190); failure to indicate game titles, which were needed to identify genres (*n* = 104); technical error with the procedure (*n* = 8); failure in the double attention check (*n* = 113). Subsequently, a total of *N* = 205 participants were left in the sample. Our participants were between 13 and 70 years old (*M* = 30.26, *SD* = 12.74), including 133 women (64.9%), 70 men (34.1%), one non-binary person, and one participant who refused to state their gender.

### Measures

#### Gaming involvement

The Gaming Involvement Scale was constructed to measure time spent on six different manifestations of gaming-related activity: gaming itself, watching game-related videos, thinking about video games, reading game-related content, talking to other people about video games, and considering game-related purchases. The participants were asked to estimate how much time they spent on these activities during an average working day and an average weekend day. Variables were calculated to show average weekly gaming involvement. DGI was calculated using the following formula: [gaming time on average weekday * 5] + [gaming time on average weekend day * 2]. IGI was calculated similarly as the sum of hours spent weekly on the gaming-related activities listed above. The tool is included in the Supplementary materials.

#### Game genres

The authors of earlier studies operationalized game genres based on the respondents’ classification of games to genres. However, most modern titles are difficult to classify unambiguously into one genre, so two people playing the same game may classify its genre differently. In the first study, we decided to minimize this uncertainty by asking the participants directly about the titles of the games they had played in the previous 2 weeks, and we then classified them ourselves. To determine the preferred genres, we asked the participants to list the games they had played in the previous 2 weeks and estimate how many hours they had spent playing each one. The responses were then categorized into 16 game genres by two independent assessors. The genres were: action-adventure, MOBA, shooters, card games, strategy games, RPG, sport games, simulations, puzzle/logical games, MMORPG, driving, battle royale, survival horror, platform games, fighting, and other games.

#### Gaming disorder risk

The Gaming Disorder Test (33) is a four-item assessment tool dedicated to screening GD symptoms according to the International Classification of Diseases, 11^th^ Revision. The study used a Polish version developed by Cudo et al. (7), which relates to the Polish cultural context. The items were rated on a 5-point Likert scale. Total scores can range from 4 to 20, with higher scores indicating greater GD risk. We treated the obtained results as a continuous variable. The internal consistency was acceptable (Cronbach’s *α* = 0.85).

#### Gaming motivation

At the end of the procedure, a Polish translation of the Gaming Motivation Inventory (GMI; 34) was used for exploratory reasons. The tool is not included in the analyzes reported in this paper. The questionnaire consisted of 88 items representing 26 motives. Each item was rated from 1 (“It does not correspond at all”) to 7 (“It corresponds exactly”). Cronbach’s *α* for the scales ranged from *α* = 0.71 (Coping) to *α* = 0.94 (Financial). The Polish version with detailed results can be found in the Supplementary materials.

### Transparency and openness

All the employed methods are described above. In line with best practices, the data, code, materials (34), and the time-stamped preregistration for the study (35) are available for download at the Open Science Framework. Statistical analyzes were performed using IBM SPSS Statistics 28 software.

## Results – Study 1

We asked our participants to list the games they had been playing during the previous 2 weeks, along with the gaming time for each game. However, an absolute majority of participants listed only game titles without gaming times. Therefore, we created dichotomous variables for each genre, where 0 indicated that the participant had not been playing any games of this genre and 1 indicated that the participant had played at least one game of a given genre.

We checked Pearson’s correlations between the three main variables. The correlation between DGI and GD was positive and moderate, *r* = 0.31 (*p* < 0.001; e.g., 36, 37). The correlation between IGI and GD was very similar, *r* = 0.30 (*p* < 0.001). DGI and IGI were correlated strongly and positively, *r* = 0.51 (*p* < 0.001).

Secondly, we performed a hierarchical multiple regression which determined the association between age, gender, DGI, IGI, and each game genre with GD. Linearity was checked with partial regression plots. Homoskedasticity was assessed by Koenker’s test for heteroskedasticity (38) using the HeteroskedasticityV3 SPSS macro (39). The test statistic was 28.10, *p* = 0.107, indicating that heteroskedasticity was not present. A Durbin-Watson statistic of 2.06 indicated the independence of the residuals. There was also no evidence of multicollinearity, as assessed by tolerance values greater than 0.61 and VIF values smaller than 1.63.

We controlled for demographic variables in Step 1, DGI in Step 2, IGI in Step 3, and game genres in Step 4. Neither age nor gender significantly predicted the dependent variable. After adding DGI to the model, the percentage of explained variance increased to 6% (F-change = 15.59, *p* < 0.001). DGI significantly positively predicted GD (*β* = 0.27). IGI added in Step 3 was also a significant positive predictor of GD (*β* = 0.22) and increased the explained variance to 9%. This change was significant (F-change = 7.79, *p* = 0.006). In the last step, we added dichotomous variables representing 16 game genres. There were 2 significant predictors among the genres: RPG (*β* = 0.15) and MMORPG (*β* = 0.14). DGI was no longer significant after adding the genres. Detailed results are presented in Table 1.

**Table 1:** Regression analysis of gaming disorder in Study 1.

	*B*	*SE*	*β*	*t*	*p*
Model 1, *F* (2,198) = 0.03; *p* = 0.972; *R*^2^_adj._ = −0.01					
(Constant)	7.27	0.60		12.14	<0.001
Age	0.00	0.02	−0.01	−0.19	0.849
Gender dichotomous	0.00	0.00	0.01	0.13	0.900
Model 2, *F* (3,197) = 5.22; *p* = 0.002; *R*^2^_adj._ = 0.06					
(Constant)	5.95	0.67		8.89	<0.001
Age	0.00	0.02	0.01	0.12	0.908
Gender dichotomous	0.00	0.00	0.03	0.39	0.699
DGI	0.07	0.02	0.27	3.95	<0.001
Model 3, *F* (4,196) = 6.00; *p* < 0.001; *R*^2^_adj._ = 0.09					
(Constant)	5.48	0.68		8.07	<0.001
Age	0.01	0.02	0.04	0.54	0.588
Gender dichotomous	0.00	0.00	0.03	0.41	0.682
DGI	0.04	0.02	0.16	2.04	0.043
IGI	0.02	0.01	0.22	2.79	0.006
Model 4, *F* (20,180) = 2.20; *p* = 0.003; *R*^2^_adj._ = 0.11					
(Constant)	5.09	0.89		5.68	<0.001
Age	0.01	0.02	0.03	0.32	0.748
Gender dichotomous	0.00	0.00	0.04	0.66	0.510
DGI	0.04	0.02	0.14	1.61	0.109
IGI	0.03	0.01	0.26	3.07	0.002
Action adventure	0.24	0.58	0.03	0.41	0.684
Battle royale	1.02	1.60	0.04	0.63	0.526
Card games	−0.67	1.17	−0.04	−0.57	0.569
Driving games	−0.49	1.19	−0.03	−0.41	0.682
Fighting games	−0.16	3.26	0.00	−0.05	0.960
MMORPG	2.22	1.12	0.14	1.98	0.049
MOBA	−0.02	0.68	0.00	−0.02	0.982
Other	0.29	0.66	0.03	0.44	0.659
Platform games	0.01	0.87	0.00	0.02	0.988
Puzzle ang logic games	1.09	0.62	0.14	1.75	0.081
RPG	1.62	0.80	0.15	2.03	0.044
Shooters	0.02	0.68	0.00	0.03	0.976
Simulations	−0.51	0.55	−0.07	−0.93	0.353
Sport games	−0.89	1.03	−0.06	−0.86	0.391
Strategy games	1.04	0.70	0.11	1.48	0.140
Survival horrors	−1.85	1.44	−0.09	−1.28	0.200

Dependent variable – Gaming Disorder.

A post-hoc power analysis of the regression analysis revealed an observed statistical power of 0.9957 (or 99.57%), indicating a very high probability of correctly detecting true relationships between the predictors and the dependent variable (40). With such a high post-hoc power, the study was well-powered, thus suggesting that the sample size was more than sufficient to detect the investigated effects.

We also tested for the moderation effect described in H5. Using the PROCESS 4.1 macro, Model 1 (41), we checked if IGI was a moderator of the relationship between DGI and GD. There was no significant interaction effect (*R*^2^ = 0.00, *F* (1, 197) = 0.04, *p* = 0.837).

## Discussion – Study 1

The aim of Study 1 was to verify five hypotheses regarding the relationship between direct and indirect gaming involvement and the risk of GD. Three of them were confirmed. Positive and moderate correlations among DGI and IGI and GD were confirmed by correlation analysis. The moderate relationship between DGI and GD risk obtained in our study did not differ from the results obtained by other authors (4, 7–10). As expected, a similar relationship was observed between IGI and GD risk; to our knowledge, this was the first attempt to study this.

The next two hypotheses, H3 and H4, were tested using regression analysis. We confirmed that adding IGI to the model, alongside DGI, significantly increased the explained variance and improved the model fit. This means that in order to estimate the risk of GD, one should consider game-related activities other than gaming itself. However, the percentage of explained variance remained at a relatively low level. We tested H4 based on our prediction that IGI would in fact enhance the relationship between DGI and GD. The results did not confirm the predicted interaction between DGI and IGI in predicting GD. Thus, we did not find evidence that the intensity of activities indirectly related to gaming in non-gaming time contributes to an increase in the harmful effects of gaming. This is more confirmation that mere involvement in gaming, also in the form of activities indirectly related to gaming, is not sufficient to fully explain risk of GD.

In order to test H5, we added game genres to the model. Two of the seven genres we mentioned in H5 turned out to be significant predictors of GD: RPG and MMORPG. Thus, we partially confirmed the hypothesis that some game genres may contribute more to the development of GD than others. It should be emphasized that our results are in line with those of previous studies. This was demonstrated, for example, by Na and colleagues for MMORPG (29) and Elliot and colleagues for RPG (24). On the other hand, as in the case of previous studies, it is difficult to identify a consistent pattern. From study to study, some genres come and go as a particular risk factor for GD.

The model fit of the final regression model was relatively poor (adj. *R*^2^ = 0.11), which could be caused by the potential limitations of the study. Firstly, the study was carried out online on a survey platform that is available to anyone interested in earning money by completing surveys. As a result, our sample included respondents from a wide age range (13–70) that were beyond the researcher’s control, as is a feature of online research. These two factors could potentially weaken the observed effects. Secondly, the incomplete data caused by the respondents’ unwillingness to provide gaming times forced us to create dichotomous variables representing genres, which could potentially distort the role of game genre.

Considering the limitations identified, we made several modifications to enhance the procedure for Study 2. To ensure data quality, we opted to conduct Study 2 offline in a controlled lab environment, specifically targeting young adults as respondents. In Study 1, we employed a labor-intensive approach to determine participants’ preferred game genre. They were asked to list the games they played the most and estimate their playing time over the past 2 weeks. Two assessors then assigned genres to the collected titles. While this approach aimed to align with actual behavior and minimize the risk of assigning one title to multiple genres based on participants’ opinions, we encountered challenges. Participants found it difficult to determine the titles, and only a few provided accurate gaming times. As a result, we had to reduce the genre variable to a dichotomous scale. Taking these factors into consideration, we decided to return to a popular method of determining genre preferences in Study 2. This method involves categorizing genres based on the percentage of playing time allocated to each genre, along with providing several examples for clarification, following the approach used by Király and colleagues (42). These adjustments aim to streamline and enhance the categorization process for genre preferences.

## Methods – Study 2

### Participants and procedure

Participants were recruited at the Jagiellonian University campus in Krakow. The screening question was: “Do you play video games on your phone, computer, tablet, or any electronic device?”. After signing the consent form, participants completed the questionnaires on the Qualtrics platform in a research lab.

The planned sample size was 218, calculated according to the formula proposed by Tabachnick et al. (32), given that a multiple regression model would involve 21 predictors. The number differs from the number in the previous study because we extended the number of game genres. Finally, we enrolled 250 participants aged from 18 to 27 years old (*M* = 21.03, *SD* = 1.76). The sample consisted of 84 women (35.3%), 140 men (58.8%), 11 non-binary persons (4.6%), and 3 participants who refused to state their gender (1.3%).

### Measures

We used the same Gaming Involvement Scale as in Study 1, followed by a modified version of the game genre classification procedure. In Study 2 we asked the participants to divide 100% of their gaming time in the last 2 weeks between the genres listed (e.g., RPG – 20%; Shooters – 80%). The game classification list for this study was based on Király et al.’s (42) video game genre list, which was extended by the genres mentioned by Matthews et al. (43). There were 18 categories listed, each one with 3–5 popular examples: RPG, MMORPG, strategy, MOBA, battle royale, shooters, action-adventure, card games, digital board games, sports games, simulations, auto-chess/auto battle games, driving, fighting, survival horror, platform games, puzzle/logic, other. We created continuous variables representing gaming time within each genre based on gaming time from the Gaming Involvement Scale. Next, we included the Gaming Disorder Test (7, 33), which obtained Cronbach’s *α* = 0.76. Lastly, we included the Gaming Motivation Inventory as in Study 1 (GMI; 34). The internal consistency of the GMI scales in Study 2 ranged from *α* = 0.64 (Coping) to *α* = 0.97 (Financial).

### Transparency and openness

All methods are described above. Statistical analyzes were performed using IBM SPSS 28. The data, code, materials (44), and the time-stamped preregistration for the study (45) are available for download on the Open Science Framework. Statistical analyzes were performed using IBM SPSS Statistics 28.

## Results – Study 2

First, we applied Pearson’s correlation to assess the relationship between DGI, IGI and GD. Both variables were positively but weakly associated with GD (for DGI *r* = 0.28, *p* < 0.001; for IGI *r* = 0.16, *p* = 0.016). DGI and IGI were also correlated moderately and positively, Pearson’s *r* = 0.45 (*p* < 0.001).

Next, we conducted a hierarchical regression analysis of GD. We controlled for age and gender in Step 1, IGI in step 2, and DGI divided into genres in step 3. Linear regression assumptions were checked as in Study 1. We checked for linearity with partial regression plots. Heteroskedasticity was assessed with the Koenker test, calculated with the HeteroskedasticityV3 SPSS macro (39). The test statistic of 13.80, *p* = 0.879 indicated no heteroskedasticity. The Durbin-Watson statistic (*DW* = 0.05) indicated a minor positive auto-correlation of residuals. There was also no evidence of multicollinearity, as assessed by tolerance values greater than 0.65 and VIF values smaller than 1.55.

Neither age nor gender could significantly predict the dependent variable. In Step 2, IGI was a significant predictor of GD (*β* = 0.14) but increased the amount of explained variance to 1%. In Step 3, the predictive power of IGI was reduced to being non-significant. Gaming time could positively predict GD within three genres: RPG (*β* = 0.19), shooters (*β* = 0.16) and driving/racing games (*β* = 0.17). A post-hoc power analysis revealed statistical power equal 0.9903 (or 99.03%), again indicating a very high probability of correctly detecting the investigated relationships (see Table 2 for the details).

**Table 2:** Regression analysis of gaming disorder in Study 2.

	*B*	*SE*	*β*	*t*	*p*
Model 1, *F* (2,217) = 0.47; *p* = 0.626; *R*^2^_adj._ = −0.01					
(Constant)	5.07	2.87		1.77	0.078
Age	0.13	0.14	0.06	0.92	0.357
Gender dichotomous	0.13	0.48	0.02	0.27	0.788
Model 2, *F* (3,216) = 1.70; *p* = 0.167; *R*^2^_adj._ = 0.01					
(Constant)	5.33	2.85		1.87	0.063
Age	0.10	0.14	0.05	0.70	0.483
Gender dichotomous	0.03	0.48	0.00	0.06	0.956
IGI	0.03	0.01	0.14	2.04	0.043
Model 3, *F* (21,198) = 1.92; *p* = 0.012; *R*^2^_adj._ = 0.08					
(Constant)	6.57	2.92		2.25	0.025
Age	−0.01	0.14	−0.01	−0.09	0.925
Gender dichotomous	−0.31	0.56	−0.04	−0.55	0.584
IGI	0.01	0.01	0.04	0.53	0.595
Action adventure	0.00	0.05	0.01	0.07	0.942
Auto chess battle	0.03	0.09	0.02	0.33	0.742
Battle royale	0.16	0.12	0.09	1.35	0.177
Card games	0.12	0.07	0.12	1.72	0.087
Computer board games	0.22	0.20	0.08	1.11	0.270
Driving games	0.37	0.15	0.17	2.48	0.014
Fighting games	−0.17	0.32	−0.04	−0.54	0.587
MMORPG	0.12	0.07	0.12	1.66	0.099
MOBA	0.02	0.03	0.04	0.61	0.544
Other	0.08	0.04	0.13	1.82	0.071
Platform games	0.14	0.09	0.11	1.57	0.119
Puzzle and logic games	0.05	0.06	0.06	0.85	0.396
RPG	0.07	0.03	0.19	2.61	0.010
Shooters	0.08	0.04	0.16	2.12	0.035
Simulations	0.04	0.06	0.05	0.76	0.448
Sport games	0.08	0.10	0.05	0.79	0.428
Strategy games	0.06	0.05	0.08	1.11	0.267
Survival horrors	−0.01	0.10	−0.01	−0.14	0.889

Dependent variable – Gaming Disorder.

Again, we tested for the interaction effect between DGI, IGI and GD. Using PROCESS macro 4.1, Model 1 (41) we checked if IGI was a moderator of the relationship between DGI and GD. There was no significant interaction effect (*R*^2^ = 0.00, *F* (1, 230) = 0.45, *p* = 0.502).

## Discussion – Study 2

In Study 2, we aimed to test the same hypotheses as in Study 1. However, due to the difficulties encountered by participants in providing information about individual game titles and playing times, we opted to employ a method consistent with previous studies [e.g., (42)]. The findings of Study 2 largely supported our previous results, albeit with some exceptions for H3 and partially H5.

As was consistent with our expectations, both genre-specific gaming involvement (DGI) and overall gaming involvement (IGI) showed positive correlations with gaming disorder (GD). However, the effect sizes were small for both variables (*r* = 0.28 for DGI and r = 0.16 for IGI). Employing regression analysis similar to that used in Study 1 but with a modified game genre measurement only accounted for a minimal proportion of the variance in GD. The inclusion of IGI in the model marginally increased the explained variance by 1%, leading to the rejection of H3. Furthermore, incorporating gaming time divided into genres in the final model resulted in a further increase in explained variance, but only to a modest extent of 8%.

Contrary to our expectations, we did not find supporting evidence for the moderating role of IGI, leading to the rejection of H4. Interestingly, we identified three specific game genres that were significantly associated with an increased risk of GD. While the inclusion of RPG and shooter genres in H5 aligned with our hypotheses, the unexpected finding was the involvement of driving/racing games, which also showed a significant association with GD risk.

## General discussion

The aim of the presented research was to verify the importance of two potential dosage effect moderators for GD risk. The first effect discovered was associated with game genres. Although numerous approaches exist in the literature, there is no conclusion regarding which game genres contribute most to GD. Therefore, when approaching this issue, we hoped not so much to make a breakthrough as to add more valuable data to the ongoing discussion. The second effect discovered was associated with the potentially moderating role of IGI (indirect gaming involvement). We expected that since salience is one of the peripheral symptoms of gaming disorder (2), it would prove to be an important moderator of the relationship between time spent on gaming (DGI, direct gaming involvement) and the risk of GD. In this case, we reasoned that people who devote more time to activities indirectly related to gaming in their gaming-free time will be more susceptible to GD. In other words, the actual playing time should make more of a difference to their risk of GD if they participate more in other gaming-related activities (e.g., thinking or reading about gaming).

The two studies presented above were designed and conducted to explore the associations between gaming involvement, gaming genre preferences, and the risk of Gaming Disorder. The modifications used in Study 2 resulted from the observed limitations of Study 1. Our research demonstrated the stability of the basic relationships within GD, which suggests the existence of fundamental principles that withstand deliberate changes in specific research protocols. Both studies were pre-registered, including the five hypotheses tested, the methods of data collection, and the analyzes. Therefore, our results can be interpreted in a sufficiently broad context.

Hypothesis 1, which posited a positive and moderate association between direct gaming involvement (DGI) and GD risk was confirmed in Study 1. This finding supports previous research indicating that greater engagement in gaming activities is linked to an increased risk of developing GD. However, in Study 2, while the association between DGI and GD risk was confirmed again, the strength of the relationship was weak. This finding challenges the commonly held assumption that increased gaming time directly corresponds to a higher risk of GD. Moreover, it suggests that factors other than mere time spent gaming (e.g., individual characteristics, gaming motivations, or specific game-related features) may play an influential role in the development of GD.

The results of both Study 1 and 2 supported Hypothesis 2, which suggested a positive association between indirect gaming participation (IGI) and GD risk. These findings indicate that not only direct gaming engagement but also indirect involvement, such as watching gaming content, may contribute to an increased risk of GD. Moreover, they emphasize the importance of considering various forms of gaming involvement when assessing GD risk. Our findings are in line with previous research focused on individual components of IGI: Cabreza-Ramirez et al. (14) showed that a feeling of belonging to the Twitch community (a service mainly focused on video game streaming) plays an important role in the model of pathological game use; Ortiz de Gortari et al. showed that intrusive thoughts regarding gaming are correlated with GD (16). Interestingly, Yildiz Durak et al. (17) showed that time spent watching gaming videos on Twitch is correlated with GD, whereas time spent watching gaming streams on unspecified platforms or gaming itself is not linked to GD. Thus, our research fits into a relatively new direction of research that verifies the role of individual behaviors indirectly related to gaming in the context of GD risk. Nevertheless, to the best of our knowledge, this was the first attempt to capture the joint role of various behaviors that comprise IGI, thus these findings are the first to show that when assessing the risk of GD, one should look at IGI as a whole in addition to simply asking participants how much time they spend on gaming itself.

The hypothesis regarding the inclusion of IGI in the hierarchical multiple regression model (H3) was confirmed in Study 1, but not in Study 2. In Study 1, the inclusion of IGI alongside DGI significantly improved the explanatory power of the model, accounting for more variance in GD risk. However, Study 2 did not replicate this finding, suggesting that the influence of IGI on GD risk may vary across different populations or contexts [see the partially conflicting results quoted above (14, 16, 17)]. It is worth noting that in Study 1 the sample was very diverse in age (13–70), and the procedure did not allow for the behavior of the respondents to be controlled when filling out the questionnaire. In contrast, in Study 2 the respondents’ group consisted of young gamers performing the survey in the laboratory. Perhaps one of these differences determined the decrease in the significance of IGI. However, Study 1 differed from Study 2 in terms of how it measured DGI. In the first case, we calculated total gaming time from the Gaming Involvement Scale; in the second case, we calculated gaming time for each genre. Considering that the absolute importance of IGI was demonstrated in both studies and only the relative importance (considering DGI) was confirmed in one of the studies, it cannot be ruled out that the operationalization of DGI could have played a key role in suppressing the importance of IGI within the DGI context. Future research on the role of IGI should be carefully designed with this issue in mind.

Hypothesis 4, which proposed that IGI would moderate the relationship between DGI and GD risk, was not confirmed in either Study 1 or Study 2. These results suggest that the interaction effect between IGI and DGI does not influence GD risk. The source of our predictions in this case was the older understanding of gaming disorder that is proposed in DSM-5 (Internet Gaming Disorder, IGD; 20). In this case, one of the nine GD criteria was preoccupation. Subjects with IGD would exhibit preoccupation with online gaming, even during other activities, at least three times a week. Building on this, we predicted that subjects who showed a high intensity of behavior directly related to gaming in combination with intense preoccupation during non-gaming time would be particularly at risk of GD. However, in order to be in line with the latest WHO guidelines on GD (46), we omitted the fact that the tool used in our study ignores the issue of preoccupation (GDT, 33). Moreover, our hypothesis seemed to fit the updated version of the I-PACE model (19), which includes environmental context (for example, medium characteristics) among the factors that potentially interact. We predicted that there would be an interaction between gaming time (DGI) and other activities (IGI) for people using not only games but also gaming content delivered through other means to satisfy their needs. This hypothesis was not supported. However, the I-PACE model does not directly postulate that the environmental factor must strictly interact – as understood through statistical moderation – with gaming. Given this and the confirmation of Hypothesis 2, it should be considered that our results confirm the general idea behind the model, which says that extending the possibility of “decisions to behave in a specific way” to include further gaming-related activities may be conducive to obtaining gratification and thus contribute to the development of GD, understood as a process. Therefore, there is a possibility that the consistent lack of moderation predicted by us is due to the tool used or to the other role of IGI in the process of GD development. This may only be resolved in future research in which one of the scales based on the IGD definition is used. Future research may also confirm the insignificance of this interaction and identify other key psychological moderators in this area linked to cognition, execution, personality, and emotions.

Regarding Hypothesis 5, which explored the association between gaming times for different genres and GD risk, the findings were partially confirmed in both studies. We found GD risk to be significantly predicted by MMORPG and RPG in Study 1, and by driving games, RPGs and shooters in Study 2. Notably, our research findings demonstrated remarkable consistency in the results for Role-Playing Games (RPGs). This consistency suggests that RPGs possess certain inherent characteristics that are significantly associated with GD outcomes. However, it is impossible to ignore the fact that one genre (driving) that was identified in our data as a significant predictor has not appeared in any previous reports known to us. In this case, we recommend caution. Given the numerous previous studies, such isolated occurrences should be treated as potential artifacts that need to be confirmed in the future. Further research is needed in this case, perhaps including a meta-analysis. A second possible approach could be to completely change the way we look at game characteristics, such as departure from the traditional but somewhat anachronistic division into genres towards the identification of specific features of games that favor GD. The foundations for such an approach have been laid, among others, by Li (47), who identified 29 genres and correlated tags with them that corresponded to the features of games. Perhaps tags or otherwise-defined characteristics might have a better chance of accurately indicating GD predictors.

## Strengths and limitations

The current paper’s strengths are (1) it consists of two studies that test the same hypotheses using partially different operationalizations, (2) pre-registration of both procedures, (3) it draws attention to a potential new explanatory variable, IGI (indirect gaming involvement), and (4) the use of two different ways of collecting data (online and in the laboratory). The presented research also has some limitations, including (1) the sample was limited to speakers of one language from a relatively homogeneous society, (2) a low percentage of people with a clear risk of GD (while corresponding to the general population), and (3) the use of only one measure of GD, which does not explicitly take into account the preoccupation criterion. The study adopted a cross-sectional design, thereby limiting the ability to derive causal conclusions from its outcomes.

## Conclusion

Gaming time alone is not the sole predictor of GD risk. Our studies, in line with previous research, found that game genre can moderate this relationship, specifically highlighting the role of RPGs. However, considering the multitude and variability of game genres, we propose a shift in focus towards analyzing game content, mechanics, and tags, as these could provide more specific and informative insights. This approach could address spatial and interpretational challenges associated with genre classification, ultimately improving the accuracy of predictive models.

Additionally, our research is the first to demonstrate that indirectly related gaming activities contribute to the variability in GD risk. These activities encompass watching game-related videos, thinking about video games, reading game-related content, discussing video games with others, and considering game-related purchases. Given that our assessment of GD risk utilized a tool based on the latest definition by the World Health Organization (46), which does not consider preoccupation, these findings are particularly significant.

Moreover, our studies show that the risk of GD can also be predicted by the extent of IGI. To gain deeper insights, further research exploring the moderating role of game characteristics and the role of non-gaming activities on the development of GD is needed. Longitudinal studies could play a pivotal role in this regard.

Meanwhile, it is crucial for clinical psychologists to consider the broader context of gaming activities and genres when assessing individuals with GD. This comprehensive perspective will allow for a more accurate evaluation and appropriate interventions.

## Data availability statement

The datasets presented in this study can be found in online repositories. The names of the repository/repositories and accession number(s) can be found at: https://osf.io/h7s3c/, https://osf.io/dkny4/.

## Ethics statement

The studies involving humans were approved by the study was carried out in accordance with Declaration of Helsinki and approved be the Institutional Research Ethics Committee of the Jagiellonian University (acceptance no. 102/2021). All subjects were assured of the right to refuse or terminate their participation at any time, confidentiality and anonymity. All participants and legal guardians of minors provided informed consent. The studies were conducted in accordance with the local legislation and institutional requirements. Written informed consent for participation in this study was provided by the participants' legal guardians/next of kin.

## Author contributions

PS: conceptualization, formal analysis, funding acquisition, methodology, project administration, and writing – initial draft. PK: investigation and writing – review. JS: conceptualization, methodology, and writing – review. PDS: investigation. SZ: computation, investigation, methodology, resources, writing – initial draft, and writing – review. AW: investigation. AS: funding acquisition and project administration. AZ: data curation, formal analysis, project administration, and writing – initial draft. All authors contributed to the article and approved the submitted version.

## Funding

This research is a part of SONATA Bis-11 research project “The transformation process from gaming involvement to gaming disorder: Delineating social and motivational antecedents from consequences,” no. 2021/42/E/HS6/00068, funded by The National Science Center (NCN) and carried out at the Faculty of Management and Social Communication, Jagiellonian University, Kraków, Poland.

## Conflict of interest

The authors declare that the research was conducted in the absence of any commercial or financial relationships that could be construed as a potential conflict of interest.

## Publisher’s note

All claims expressed in this article are solely those of the authors and do not necessarily represent those of their affiliated organizations, or those of the publisher, the editors and the reviewers. Any product that may be evaluated in this article, or claim that may be made by its manufacturer, is not guaranteed or endorsed by the publisher.
