# Supplementary material for: It’s not just about how long you play. Indirect gaming involvement and genre preferences in predicting gaming disorder risk: evidence from preregistered studies
Source: Front Psychiatry. 2023 Sep 18;14:1230774. doi: 10.3389/fpsyt.2023.1230774 (PMC10546925; doi:10.3389/fpsyt.2023.1230774)
Supplement: Supplementary file 1 [file Table_1.docx]

Supplementary Material

It's not just about how long you play. Indirect gaming involvement and genre preferences in predicting Gaming Disorder risk: evidence from preregistered studies

Paweł Strojny^1^, Patrycja Kiszka^1^, Jolanta Starosta^1^, Paulina Daria Szyszka^1,2^, Sylwia Zegar^3^, Anna Winiarska^3^, Agnieszka Strojny^1^, Aleksandra Zajas^1,3*^

^1^Institute of Applied Psychology, Faculty of Management and Social Communication, Jagiellonian University, Kraków, Poland

^2^Institute of Psychology, Faculty of Philosophy, Jagiellonian University, Kraków, Poland

^3^ Doctoral School in the Social Sciences, Jagiellonian University, Kraków, Poland

*** Correspondence:**Corresponding Author
aleksandra.zajas@uj.edu.pl

**Supplementary Table 1**

Gaming Involvement - The scale of involvement in video games

| How many MINUTES a day do you spend on the following activities? | | |
| --- | --- | --- |
|  | On a working day | On a weekend day |
| 1. Playing video games |  |  |
| 2. Thinking about video games |  |  |
| 3. Reading guides, tutorials, reviews, theories, extra lore related to video games |  |  |
| 4. Watching video game streams and gameplays (including esports) |  |  |
| 5. Talking or texting about video games |  |  |
| 6. Considering buying additional content and collectibles related to games (skins, gamepasses, ingame currency, figurines, t-shirts) |  |  |

**Supplementary Table 2**

GMI scales reliability – Study 1

| Scale name | Cronbach’s alpha |
| --- | --- |
| Advancement | .86 |
| Amotivation | .74 |
| Autonomy | .86 |
| Boredom | .82 |
| Competence | .81 |
| Competition | .82 |
| Completion | .86 |
| Coping | .71 |
| Escape | .87 |
| Exploration + Mechanics | .88 |
| Fantasy | .85 |
| Financial | .94 |
| Game skills | .89 |
| Identity | .88 |
| Introjected regulation | .84 |
| Recreation | .81 |
| Skill development | .91 |
| Social | .89 |
| Status | .84 |
| Arousal-action | .89 |
| Cooperation | .92 |
| Customization | .91 |
| Destruction | .89 |
| Graphics | .89 |
| Story | .88 |
| Strategy | .89 |

**Supplementary Table 3**

GMI scales reliability – Study 2

| Scale name | Cronbach’s alpha |
| --- | --- |
| Advancement | .87 |
| Amotivation | .81 |
| Autonomy | .83 |
| Boredom | .86 |
| Competence | .77 |
| Competition | .83 |
| Completion | .79 |
| Coping | .64 |
| Escape | .91 |
| Exploration + Mechanics | .88 |
| Fantasy | .84 |
| Financial | .97 |
| Game skills | .86 |
| Identity | .80 |
| Introjected regulation | .68 |
| Recreation | .81 |
| Skill development | .84 |
| Social | .87 |
| Status | .80 |
| Arousal-action | .88 |
| Cooperation | .95 |
| Customization | .95 |
| Destruction | .85 |
| Graphics | .96 |
| Story | .94 |
| Strategy | .90 |

**Supplementary Table 4**

Pearson’s correlation matrix of variables used in regression analysis with means and standard deviations of continuous variables from Study 1

|  | *M* | *SD* | 1. | 2. | 3. |
| --- | --- | --- | --- | --- | --- |
| 1. GD | 7.21 | 3.23 | - | **.31^***^** | **.30^***^** |
| 2. DGI | 16.75 | 13.07 | **.31^***^** | - | **.51^***^** |
| 3. IGI | 31.86 | 31.61 | **.30^***^** | **.51^***^** | - |
| 4. Action_adventure | - | - | .07 | .12 | .10 |
| 5. Battle royale | - | - | .07 | .08 | .01 |
| 6. Card games | - | - | -.10 | .01 | -.02 |
| 7. Driving games | - | - | .01 | .11 | .08 |
| 8. Fighting games | - | - | .00 | .05 | .00 |
| 9. MMORPG | - | - | **.15^*^** | .06 | .03 |
| 10. MOBA | - | - | .06 | **.23^***^** | **.26^***^** |
| 11. Other | - | - | .00 | .06 | -.04 |
| 12. Platform games | - | - | .00 | .00 | .00 |
| 13. Puzzle and logic games | - | - | .04 | -.14 | **-.21^**^** |
| 14. RPG | - | - | **.19^**^** | **.18^*^** | **.15^*^** |
| 15. Shooters | - | - | .01 | .06 | .05 |
| 16. Simulations | - | - | -.05 | .02 | .13 |
| 17. Sport games | - | - | -.06 | .07 | .02 |
| 18. Strategy games | - | - | .05 | .01 | -.10 |
| 19. Survival horrors | - | - | -.06 | **.16^*^** | -.02 |

*Note.* ^***^ - *p* < ,001; ^**^ - *p* < .01; ^*^ - *p* < .05. DGI and IGI measurement units were hours per week.

**Supplementary Table 5**

Pearson’s correlation matrix of variables used in regression analysis with means and standard deviations of continuous variables from Study 2

|  | *M* | *SD* | 1. | 2. | 3. |
| --- | --- | --- | --- | --- | --- |
| 1. GD | 7.88 | 3.44 | - | **.28^***^** | **.16^*^** |
| 2. DGI | 11.99 | 8.29 | **.28^***^** | - | **.45^***^** |
| 3. IGI | 17.45 | 18.41 | **.16^*^** | **.45^***^** | - |
| 4. Action adventure | 2.09 | 4.73 | .00 | **.28^***^** | **.28^***^** |
| 5. Auto chess battle | .48 | 2.56 | .00 | .13 | .13 |
| 6. Battle royale | .32 | 1.93 | .10 | **.18^**^** | .07 |
| 7. Card games | 5.23 | 64.78 | -.07 | .07 | -.09 |
| 8. Computer board games | .24 | 1.17 | .08 | .03 | -.07 |
| 9. Driving games | .34 | 1.59 | **.17^**^** | **.14^*^** | .04 |
| 10. Fighting games | .11 | .69 | -.07 | -.02 | -.01 |
| 11. MMORPG | .69 | 3.27 | .08 | **.20^**^** | .03 |
| 12. MOBA | 2.85 | 8.31 | -.02 | **.35^***^** | .08 |
| 13. Other | 1.03 | 3.97 | .07 | **.24^***^** | .10 |
| 14. Platform games | .68 | 2.55 | .11 | **.17^**^** | .03 |
| 15. Puzzle and logic games | 6.42 | 64.99 | -.07 | .07 | -.12 |
| 16. RPG | 9.05 | 65.16 | .04 | **.46^***^** | **.28^***^** |
| 17. Shooters | 2.40 | 6.62 | **.14^*^** | **.27^***^** | **.23^***^** |
| 18. Simulations | 1.46 | 4.14 | .04 | **.16^*^** | .00 |
| 19. Sport games | .83 | 3.03 | -.01 | **.15^*^** | -.04 |
| 20. Strategy games | 5.76 | 64.80 | .02 | **.22^***^** | **.24^***^** |
| 21. Survival horrors | .48 | 2.21 | .01 | **.13^*^** | **.15^*^** |

*Note.* ^***^ - *p* < ,001; ^**^ - *p* < .01; ^*^ - *p* < .05. Measurement units were hours per week.
